# Carbonized PBO-Encapsulated Plasma-Activated Carbon Fibers Enabled Enhanced Thermal Conductivity and Mechanical Properties

**DOI:** 10.3390/ma19102105

**Published:** 2026-05-16

**Authors:** Xiaohui Zhang, Guangsheng Huang

**Affiliations:** 1 College of Materials Science and Engineering, Chongqing University, Chongqing 400044, China; zxheart@126.com; 2 Liaoning Xinghui Carbon Materials Technology Co., Ltd., Shenyang 110819, China

**Keywords:** carbon fibers, PBO, plasma treatment, carbonization, mechanical property, thermal insulation

## Abstract

Application of polyacrylonitrile-derived carbon fiber (CF) as a thermal insulation material is restricted by inherently high thermal conductivity. Encapsulation of poly(p-phenylene benzobisoxazole) (PBO) on CF was supposed to improve the mechanical and heat resistance of CF, which would be desired to improve mechanical and thermal-insulating performances. In this work, PBO molecules were uniformly coated onto the surface of air plasma-treated CF. Carbonized PBO-encapsulated CF (CF@CPBO) was prepared via thermal treatment at 600–1400 °C. At higher carbonization temperatures, CF@CPBO exhibited a cleaner surface, more radial graphite layers within fibers, enhanced crystallinity of carbon layers (amorphous to 0.337 nm of interplanar spacing), reduced defective/graphitic content (0.959–0.909 of I_D_/I_G_), decline in surface O content (20.1–9.6 at.%) and improved symmetry of the C-C deconvoluted peak. After weaving them into a net and compression molding, CF@CPBO felts with a random distributed structure (no voids and no fiber bundles) presented improved compression strength (10.5–25.6% of enhancement than unmodified CF) and excellent compression-recovery performance (130.9–110.8 MPa) through 10 cycles. Thermal conductivity values of CF@CPBO felts at 30–1800 °C were 0.13–1.42 W/m/K, which were 42.2–62.6% of unmodified CF. This work proposes an efficient strategy for regulating the high-performance organic fiber structure through heat treatment-induced processes.

## 1. Introduction

During the last decade, the proportion of carbon fiber (CF)-reinforced composites in aircraft structural materials has kept increasing. Their characteristics including light weight, high specific strength, high specific stiffness, and ease of processing are conducive to increasing the flight speed and flight range of aircrafts [1,2]. Taking polyacrylonitrile (PAN)-based CF as an example, PAN-based CF is the most widely used high-performance CF in the current market, accounting for over 90% of the total CF production. PAN-based CF has low production costs, high tensile strength and modulus, and low thermal expansion coefficient. Due to its high specific strength and specific stiffness, PAN-based CF is often used in the load-bearing components of spacecraft and aircraft, which can meet the lightweight requirements in the aerospace field [3]. The low thermal expansion coefficient of PAN-based CF enables composite materials with high dimensional stability, thus satisfying the needs of aerospace vehicles for use in extreme environments. The melting point of PAN-based CF is as high as 3650 °C. In inert gases or vacuum environments, its temperature resistance can reach above 2000 °C. This enables CF to maintain the structural integrity and thermal conductivity effect even under extreme high-temperature conditions, which is superior to many traditional thermal conductivity materials [4,5].

The friction between the outer structural components and the air during high-speed flight of aircrafts would generate a large amount of heat. It is an urgent issue to decrease the thermal conductivity of CF-based structural materials to isolate the heat outside the components of an aircraft, thereby protecting important systems from damage and ensuring the safety of the aircraft [6,7]. However, PAN-based CF belongs to an anisotropic large-sized graphite material. The phonon and electron transmission efficiency of PAN-based CF graphite materials is high. The axial thermal conductivity of CF is favorable. Thus, the prevention of heat conduction in CF would be a desired approach to enable the thermal insulation of CF composite materials. Moreover, the density of CF usually ranges from 0.1 to 0.5 g/cm^3^, which is much lower than that of metals and other ceramic materials [8,9]. Despite the low density of CF, asphalt-based and resin-based CFs in the heat-insulation market still suffer from weak mechanical properties when used as structural materials for aircraft. Therefore, the development of novel CF composite materials with combined low thermal conductivity and excellent mechanical properties would be highly desired.

As aromatic heterocyclic rigid rod-shaped polymers, poly-p-phenylene benzobisoxazole (PBO) fibers with highly oriented molecular chains along the fiber axis present excellent mechanical properties, high thermal stability and flame retardancy [10,11,12]. The combination of PBO with CF can inherit the high orientation characteristic of the mother fiber and form graphite layers that are closely stacked along the fiber axis during the carbonization and graphitization processes. For instance, PBO fibers would carbonize into a disordered graphite structure above 1600 °C and achieve perfect graphite-like layers above 2300 °C. This structure could achieve a higher crystallinity during the graphitization process at higher temperatures [13,14]. Kaburagi et al. observed that PBO and CF composites exhibited radial radiating and open wedge-shaped morphologies similar to those of intermediate-phase asphalt-based CF. The large-sized graphite layers in PBO/CF composite were tightly stacked radially along the fiber axis. The formation of wedge-shaped splitting morphology was caused by the stress concentration within the fibers and the release of gases during the heating treatment process, which seriously damaged the mechanical properties of the fibers [15,16].

Given the poor interfacial bonding ability of PBO molecules with CF induced by the absence of active groups in rigid rod-like PBO molecules, it is necessary to combine PBO molecules with CF more effectively. The low-temperature plasma technique can significantly enhance the bonding strength of the CF surface without affecting the strength of the fibers themselves [17,18]. Impurities and oxide layers could be removed via plasma treatment, and active groups such as hydroxyl (-OH), carbonyl (-COOH), and amino (-NH_2_) could be introduced onto the surface of CF to increase the surface polarity and enhance the affinity with other matrix, thereby strengthening the interfacial bonding strength [19,20]. Moreover, microstructure changes including the formation of micro-undulations and an increase in nanoscale roughness may be induced. These structural changes can increase the surface area and contact area with other materials to facilitate the interfacial bonding.

In this work, PAN-based CF was treated via the low-temperature air plasma. Then, PBO molecules were uniformly coated onto the surface of treated PAN-based CF. PBO-coated CF (CF@PBO) was carbonized in the nitrogen atmosphere at three temperatures including 600 °C, 900 °C and 1400 °C. Morphology, crystalline structure, defective/graphitic structure in the matrix and chemical states of surface elements of carbonized CF@PBO (CF@CPBO) were systematically characterized using multiple techniques. After weaving them into a net and compression molding, the compression-recovery mechanical properties of CF@CPBO felts were evaluated at 40%, 60% and 80% of strains through 10 cycles. Furthermore, thermal conductivity values of CF@CPBO felts at 30–1800 °C were determined. The relationship between PBO-derived carbon and various graphitized structures and mechanical/thermal conductivity properties was fully discussed.

## 2. Materials and Methods

### 2.1. Materials

PAN-based CF (M50J, 14 μm in diameter, 1.83 g/cm^3^ in density, 4.70 GPa of tensile strength, 451 GPa of tensile modulus and 1.04% of tensile elongation) was obtained from Chongqing University (Chongqing, China). PBO fiber (PBO-AS, 18 μm in diameter, 1.53 g/cm^3^ in density, 5.36 GPa of tensile strength, 167 GPa of tensile modulus and 3.55% of tensile elongation) was purchased from Institute of Chemistry, Chinese Academy of Sciences (Beijing, China). All chemicals and reagents were of analytic grade and used directly without further treatment.

### 2.2. Preparation of CF@CPBO Felt

#### 2.2.1. Encapsulation of CF by PBO via Plasma Treatment

**Figure 1:**
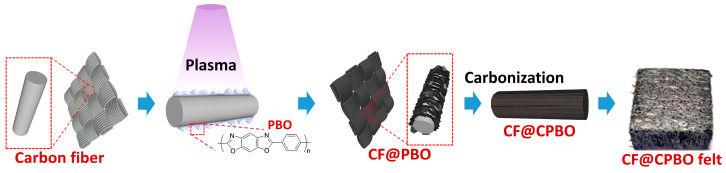
Preparation illustration of CF@CPBO felt.

PAN-based CF was treated via a radiofrequency plasma in air before the grafting of PBO (Figure 1). Specifically, PAN-based CF was washed with deionized water and dried thoroughly. A total of 1.0 g PAN-based CF was spread out on the culture dish uniformly and the culture dish was placed in the plasma chamber. The radiofrequency power in a range of 0–500 W was generated by a 13.56 MHz generator. Then the air was inflated into the chamber by keeping the pressure at 50.0 Pa. The air plasma treatment was performed for 10.0 min at 300 W. Meanwhile, PBO polymer was ultrasound-dissolved in methylsulfonic acid at 80 °C. After the plasma treatment, plasma-etched PAN-based CF was added into the PBO dispersion solution at 80 °C with continuous stirring for 6 h. As the coating of PBO on CF was achieved, CF@PBO was rinsed by deionized water to remove methylsulfonic acid. Then CF@PBO was dried at 60 °C for further usage.

#### 2.2.2. Preparation of CF@CPBO Felts

Firstly, CF@PBO was carbonized to be CF@CPBO. CF@PBO was heated in the tube furnace for 10 min with the protection of N_2_ flow (100 sccm). The heating temperature was set as 600 °C, 900 °C and 1400 °C, respectively. According to the heating temperature, obtained CF@CPBO products were termed as CF@CPBO-600, CF@CPBO-900 and CF@CPBO-1400, respectively. Then, carbonized CF@CPBO was combed into a net via a cross-plane wave method. CF was interweaved layer upon layer. The face density was 50 g/m^2^ with a twisting tension of 50 N. Finally, the net was pressed under 10 MPa with a pressing rate of 0.5 MPa/min at room temperature. CF@CPBO felts including CF@CPBO-600 felt, CF@CPBO-900 felt and CF@CPBO-1400 felt were fabricated via compression molding for 10 min.

### 2.3. Characterization

Morphologies of initial PAN-based CF, plasma-treated PAN-based CF, PBO-coated CF, and carbonized CF@PBO were observed using scanning electron microscopy (SEM, Hitachi S4800, Tokyo, Japan) and transmission electron microscopy (TEM, JEOL JEM-2100F, Tokyo, Japan). The crystal phase and crystalline structure were analyzed via X-ray diffraction (XRD, Bruker D8 Advance, Karlsruhe, Germany). Defective and graphitic structures were recorded via a laser confocal Raman spectrometer (Renishaw inVia, Gloucestershire, UK) equipped with a 24.3 mW Ar laser at 532 nm. Chemical states of surface elements were analyzed via X-ray photoelectron spectroscopy (XPS, Shimadzu Axis Ultra DLD, Kyoto, Japan).

### 2.4. Mechanical Property and Thermal Conductivity Measurement

Compression stress–strain curves of CF@CPBO felt with three compression strain contents (i.e., 40%, 60% and 80%) were investigated. Nine samples for each CF@CPBO felt (18 mm length × 18 mm width × 13.5 mm height) in total were divided into three groups with three samples in each group. Independent compression tests were conducted on each group. The compression strains were 0%-40%-0%, 0%-60%-0%, and 0%-80%-0%. The compression rate of the universal material testing device (Instron 5569A, Norwood, MA, USA) was kept consistent at 1 mm/min for all three tests. The average values of the three tests were taken to reduce the experimental error. Thermal conductivity of samples was measured via a laser flash method using a laser thermal conductivity tester (Netzsch LFA467, Selb, Germany).

## 3. Results and Discussion

### 3.1. Morphology Characterization of Plasma-Treated PAN-Based CF

**Figure 2:**
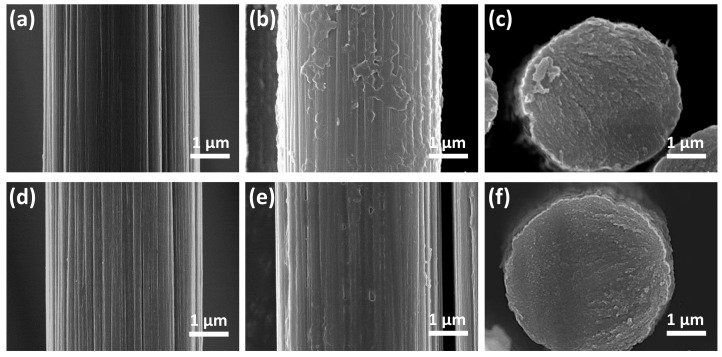
Surface morphology of untreated initial PAN-based CF (**a**). Surface and cross-sectional morphologies of PBO-coated untreated PAN-based CF (**b**,**c**). Surface morphology of plasma-treated PAN-based CF (**d**). Surface and cross-sectional morphologies of PBO-coated plasma-treated PAN-based CF (**e**,**f**).

The surface and cross-section morphologies of samples were observed. Both untreated PAN-based CF (Figure 2a) and air plasma-treated PAN-based CF (Figure 2d) exhibited longitudinal grooves distributed parallel to the fiber axis on their surfaces. Compared with untreated PAN-based CF, the grooves on air plasma-treated PAN-based CF were etched deeper and were more distinct. For PBO-coated untreated PAN-based CF, obvious PBO agglomeration occurred on the fiber surface (Figure 2b). The grafting thickness of PBO on the surface of CF was not uniform, and there were still obvious grooves on the surface of CF (Figure 2c). The surface of PBO-coated air plasma-treated PAN-based CF was uniform (Figure 2e,f). Thus, air plasma-treated PAN-based CF was applied in the following experiments, which was more conducive to the uniform grafting effect of PBO molecules. The surface of CF was uniformly coated with a thin layer of PBO without obvious agglomeration, and the thickness was uniform.

### 3.2. Characterization of CF@CPBO

**Figure 3:**
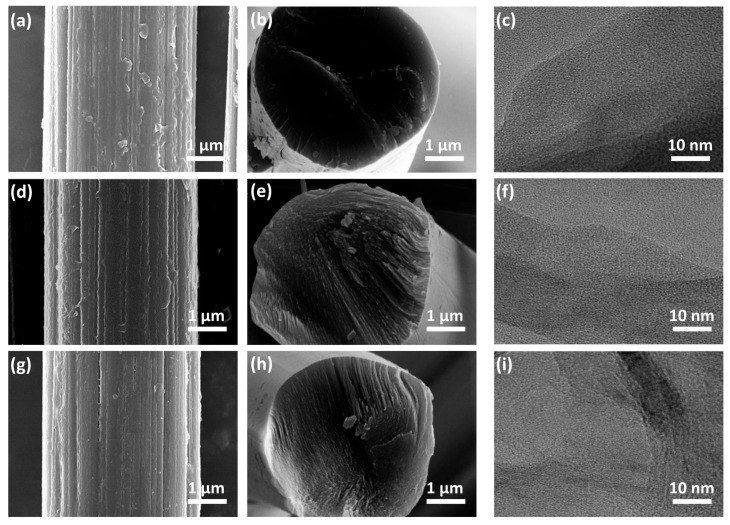
Surface morphology, cross-sectional morphology and TEM observation of CF@CPBO-600 (**a**–**c**), CF@CPBO-900 (**d**–**f**), and CF@CPBO-1400 (**g**–**i**).

SEM and high-resolution TEM were applied to observe the surface and cross-sectional morphologies of CF@CPBO carbonized at three thermal treatment temperatures (i.e., 600 °C, 900 °C and 1400 °C) and the state of graphite microcrystals within these fibers. When the carbonization degree was relatively low (e.g., 600 °C), PBO was attached obviously on the surface of CF@CPBO-600 (Figure 3a) and no radial pattern was observed on the cross-section of CF@CPBO-600 (Figure 3b). As the carbonization temperature increased to 900 °C, less PBO attachments were observed on the surface of CF@CPBO-900 (Figure 3d) and radial patterns began to appear on the cross-section of CF@CPBO-900 (Figure 3e). As the carbonization temperature increased to 1400 °C, CF@CPBO-1400 presented a relatively clean surface (Figure 3g) and a large number of radial graphite layers were generated within the fibers (Figure 3h). The cross-section of CF@CPBO-1400 fibers exhibited a radial texture similar to that of asphalt-based CF. Graphitic layers were neatly arranged along the radial direction of the fibers, while the disordered graphite structure was significantly reduced.

The high-resolution TEM image of CF@CPBO-600 (Figure 3c) exhibited an amorphous form. The pyrolyzed carbon inherited the highly oriented characteristics of the parent fiber, forming an anisotropic orientation along the fiber axis. However, the disordered graphite dominated, and no regularly arranged graphite layer appeared. As the heat treatment temperature increased to 900 °C, the preferred orientation of graphite in CF@CPBO-900 (Figure 3f) was further improved, and a small number of graphite microcrystals began to appear. Their crystal plane spacing was 0.347 nm, but a large amount of disordered structure still existed. As the heat treatment temperature increased to 1400 °C, the grain size significantly increased and layered nano-graphite microcrystalline structures appeared in CF@CPBO-900 (Figure 3i). The crystallinity of graphite layers was significantly improved, and the interplanar spacing could be reduced to 0.337 nm, gradually approaching the interplanar spacing of the ideal graphite microcrystals.

XRD analysis was carried out to analyze the graphite crystal type of CF. Typical diffraction peaks located at 15.9°, 25.7° and 27.3° were clearly exhibited, which were attributed to PBO. The diffraction peak at around 25.7° corresponding to the (002) peak of the graphite stacking direction (c-axis) was presented in XRD patterns of all PBO/CF samples. Its intensity increased rapidly with the increase in heat treatment temperature. Moreover, the position of the (002) peak shifted from 25.3° for CF@CPBO-600 to 26.3° for CF@CPBO-1400. The interlayer spacing (d_002_) and grain size (Lc) of graphite microcrystals were calculated based on the Bragg equation and Scherrer formula. The results showed that the interlayer spacing of graphite microcrystals significantly decreased with the increase in the heat treatment temperature, with 0.352 nm for CF@CPBO-600, 0.352 nm for CF@CPBO-900, and 0.339 nm for CF@CPBO-1400, which were close to the 0.335 nm value for the ideal graphite state. The stacking thickness rapidly increased (1.299, 1.791 and 19.493 nm for CF@CPBO-600, CF@CPBO-900 and CF@CPBO-1400, respectively), indicating that the more ordered graphite structure within the fibers stacked along the thickness direction. Hence, CF@CPBO with an enhanced thermal treatment temperature presented a larger graphite microcrystal size and enhanced ordered degree of layered stacking. Improvement in crystal orderability can reduce stress concentration points, enhancing the mechanical properties (such as tensile strength and fracture toughness) and thermal stability (increased interlayer bonding force, improved high-temperature deformation resistance) of CF.

**Figure 4:**
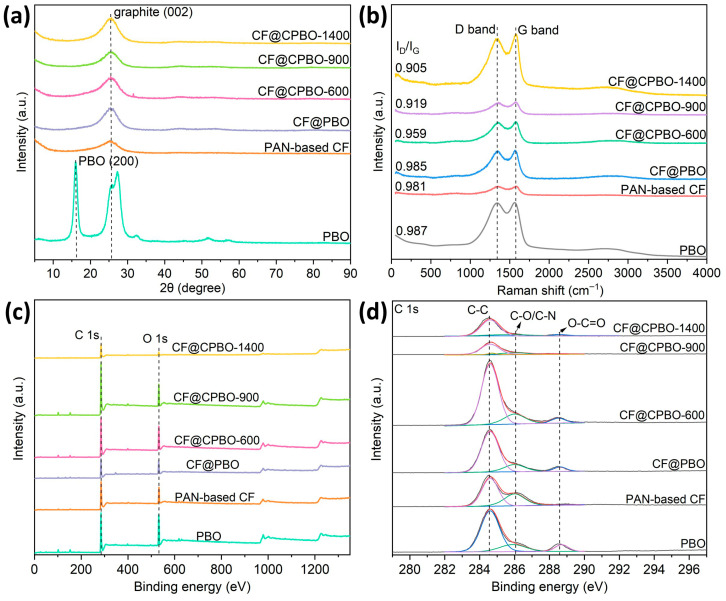
XRD patterns (**a**), Raman spectra (**b**), XPS survey (**c**) and C 1s (**d**) of PBO, PAN-based CF, CF@PBO, CF@CPBO-600, CF@CPBO-900, and CF@CPBO-1400.

Raman spectroscopy is widely used to characterize the graphitic structure and lattice vibrations of carbon materials. Band D and band G represent the lattice defects of carbon atoms and the characteristic vibration modes of sp^2^-hybridized carbon atoms, respectively. The intensity ratio of band D and band G (I_D_/I_G_) is an efficient factor to evaluate the structure of carbon materials. As illustrated in Figure 4b, typical band D and band G were both clearly exhibited in the Raman spectra of all samples. I_D_/I_G_ values were 0.987 for PBO, 0.981 for PAN-based CF and 0.985 for CF@PBO. Large numbers of defects existed in the matrix of PBO and CF@PBO. As the heating temperature of CF@PBO increased from 600 °C to 1400 °C, I_D_/I_G_ values increased continuously, with 0.959 for CF@CPBO-600, 0.919 for CF@CPBO-900, and 0.905 for CF@CPBO-1400. It is indicated that the graphitic degree of CF@CPBO increased with thermal treatment temperature, presenting less defects. Thus, carbonization of encapsulating PBO on CF could enhance the graphite structure of CF.

XPS measurement was performed to investigate the chemical states of surface elements during the carbonization process. As displayed in Figure 4c, strong C 1s and O 1s peaks were clearly exhibited in the XPS survey spectra of all samples. Specifically, the atomic ratios of O on the surfaces of samples were 23.9 at.% for PBO, 16.6 at.% for PAN-based CF and 21.0 at.% for CF@PBO (Table 1). As the heating temperature of CF@CPBO increased from 600 °C to 1400 °C, the atomic ratios of O on the surfaces decreased continuously from 20.1 at.% to 9.6 at.%. The chemical states of C in C 1s (Figure 4d) included three major components, i.e., C-C at 284.6 eV, C-O/C-N at 286.1 eV and O-C=O at 288.6 eV [21]. As the heating temperature of CF@CPBO increased, the symmetry of the C-C main peak in the C 1s spectra enhanced. The improvement in chemical purity of CF could enhance the chemical stability of CF, including resistance to oxidation and corrosion, while reducing defect sites and indirectly improving mechanical properties. Combining the above results, it is deduced that the surface of PBO-coated air plasma-treated PAN-based CF was uniformly covered with PBO molecules, which could completely cover the grooves on the surface of CF.

The oxazole ring (an oxygen-containing heterocyclic ring) in the PBO molecular chain breaks down at high temperatures. The oxygen atoms on the ring combine with adjacent carbon atoms and are released in the form of small molecular gases. When the PBO fibers are heated above 560 °C, a significant weight loss occurs, indicating a large release of oxygen-containing gases. The high-temperature carbonization provides sufficient energy for the cleavage of the oxazole ring in the PBO molecular chains, causing the oxygen atoms within them to detach in stable small-molecule forms such as CO and H_2_O, resulting in a net loss of oxygen elements on the fiber surface. This trend intensifies with an increase in temperature.

### 3.3. Morphology of CF@CPBO Felt

**Figure 5:**
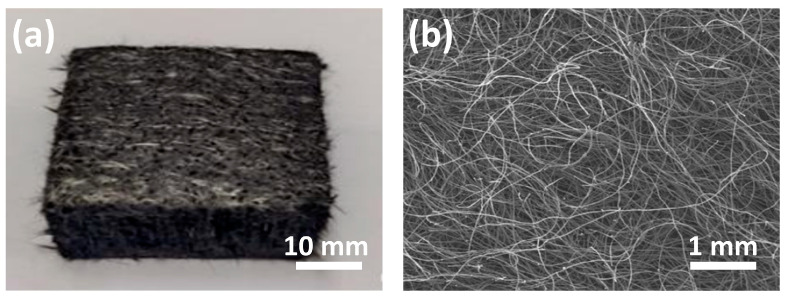
Photograph (**a**) and SEM image (**b**) of CF@CPBO felt.

CF@CPBO was woven into a net using a carding machine followed with pressing. A photograph of CF@CPBO felt is shown in Figure 5a. The pressing technique with high pressure endowed CF@CPBO felt with excellent mechanical properties and lightweight characteristics. The microscopic morphology of CF@CPBO felt shown in Figure 5b presents a random distributed structure with no voids and no fiber bundles.

### 3.4. Mechanical Property Studies of CF@CPBO Felts

**Figure 6:**
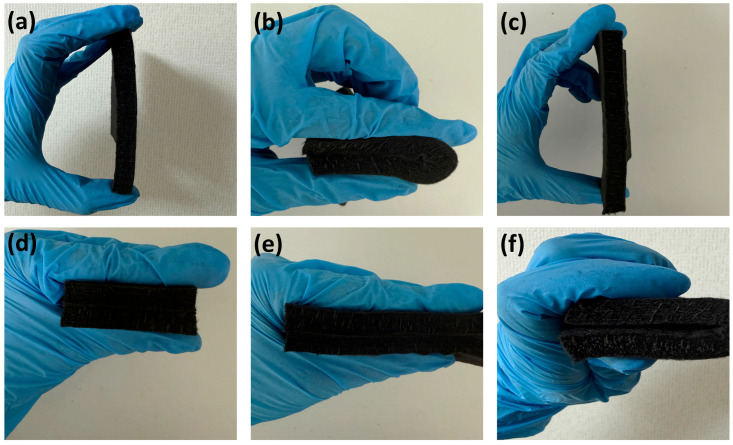
Photographs of compression-recovery test. Initial CF@CPBO felt (**a**). A 180° fold of felt (**b**). Recovery of folded felt (**c**). Initial CF@CPBO felt (**d**). Compressive CF@CPBO felt (**e**). Recovery of compressive felt (**f**).

Mechanical properties of CF@CPBO felt were visibly shown using a manual bend–compression–recovery test. Initial CF@CPBO felt could be cut into a plate-like sheet with a size of 18 mm in length × 18 mm in width × 13.5 mm in height (Figure 6a). As external force was applied, CF@CPBO felt could be bent to 180° (Figure 6b). As the external force was removed, bent CF@CPBO felt recovered totally (Figure 6c). Moreover, CF@CPBO felt could be further pressed with vertical compression force (Figure 6d,e). As the vertical compression force was removed, compressive CF@CPBO felt could be recovered (Figure 6f).

**Figure 7:**
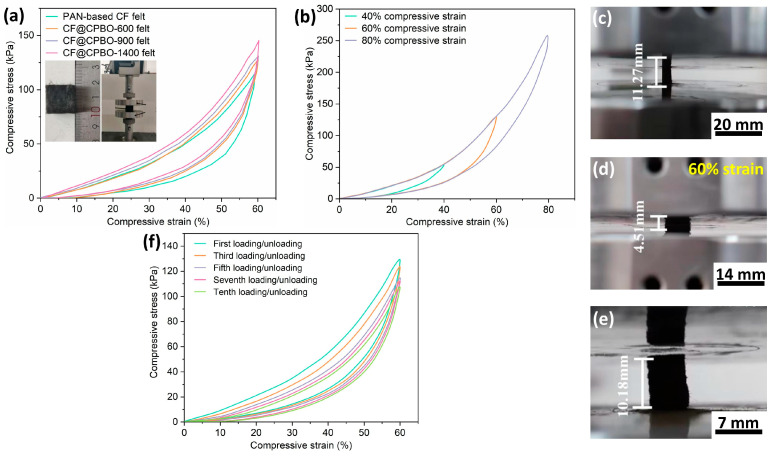
Compression stress–strain curves of CF@CPBO felt with 40%, 60% and 80% strains (**a**). Cyclic compression loading/unloading of CF@CPBO felt with 60% strain (**b**,**f**). Photographs of CF@CPBO felt before compression (**c**), at 60% strain (**d**) and after 10 cycles of loading/unloading (**e**). The compression rate was at 1 mm/min for all mechanical tests.

To accurately evaluate the compression-recovery property of CF@CPBO felt, compression stress–strain curves of CF@CPBO felts with three compression strain contents (i.e., 40%, 60% and 80%) were recorded as operated in the inset photographs of Figure 7a. PAN-based CF felt, CF@CPBO-600 felt, CF@CPBO-900 felt and CF@CPBO-1400 felt exhibited typical compression and rebound performance. At 60% of compressive strain, the compressive stresses were 145.3 MPa for CF@CPBO-1400 felt, 130.9 MPa for CF@CPBO-900 felt, 127.9 MPa for CF@CPBO-600 felt, and 115.7 MPa for PAN-based CF felt. CF@CPBO-1400 felt presented the highest compressive mechanical property. High-temperature thermal treatment of CF may exert a negative impact on the mechanical properties of the fibers. According to the Weibull weakest-link theory, the point of tensile and compression fracture is often the area where defects are concentrated [22]. Severe defects such as holes and cracks can induce the fibers to break at this location. PAN-based CF felt presented the lowest compressive strength value. After the surface grafting of PBO polymer, the compressive strength of samples at 60% of strain followed the sequence CF@CPBO-1400 felt > CF@CPBO-900 felt > CF@CPBO-600 felt > PAN-based CF felt. The strong π-π conjugation interaction between PBO and CF can effectively repair the defects on the CF surface, thereby reducing the stress concentration and fracture caused by the defects [23,24]. The increased compressive strength of CF@CPBO after higher temperature thermal treatment should be attributed to the higher crystallinity and orientation of PBO-derived carbon. It is believed that CF@CPBO would be more competitive in compressive mechanical performance and could be a new choice for low-thermal-conductivity and high-performance carbon fibers.

Moreover, by taking CF@CPBO-1400 as an example, the compression strains could be cycled as 0%-40%-0%, 0%-60%-0%, and 0%-80%-0% (Figure 7b). The maximum stresses at 40%, 60% and 80% of strain were 54.9 MPa, 130.9 MPa and 261.6 MPa, respectively. CF@CPBO felt with an initial height of 11.27 mm was located vertically with the compressive platform (Figure 7c). By taking 60% strain as an example, CF@CPBO felt was compressed to 4.51 mm (Figure 7d) and could be recovered to 10.18 mm as the compressed force was removed (Figure 7e). The compression rate was at 1 mm/min for all three tests. The average values of three tests were taken to reduce the experimental error. The compression-recovery test of CF@CPBO-1400 was carried out for 10 cycles, presenting a typical compression-rebound property. The maximum stresses at 60% of strains were 130.9 MPa for first loading/unloading, 125.7 MPa for third loading/unloading, 117.2 MPa for fifth loading/unloading, 115.6 MPa for seventh loading/unloading, and 110.8 MPa for tenth loading/unloading. This declining trend may be attributed to the brittle failure of CF induced by the cyclic loading/unloading. Permanent deformation was observed after the compression cycles. After 10 cyclic compression loadings/unloadings of CF@CPBO felt with 60% strain, residual strain (i.e., ε residual) was 4.86%. We appreciate the careful and valuable comment proposed by the reviewer.

### 3.5. Thermal Conductivity Property Studies of CF@CPBO Felts

**Figure 8:**
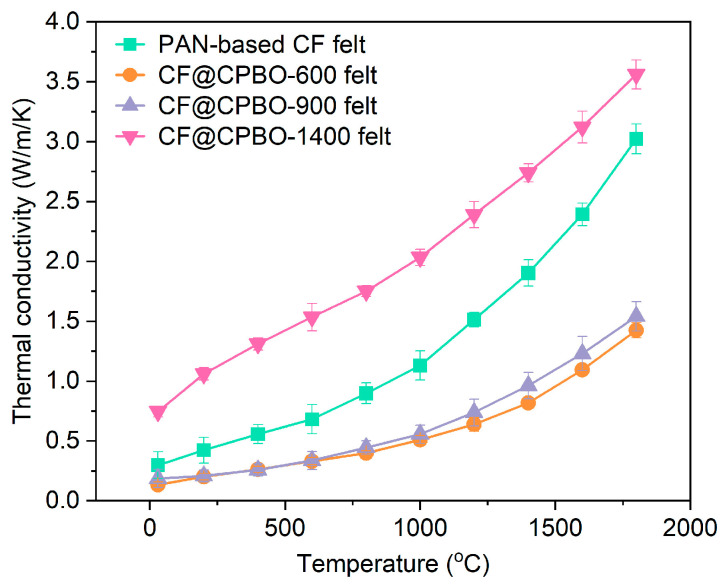
Thermal conductivity of PAN-based CF felt and CF@CPBO felts.

Thermal conductivity values of CF@CPBO felts at 30–1800 °C were determined via a laser flash method using a laser thermal conductivity tester (Netzsch LFA467, Selb, Germany). As illustrated in Figure 8, thermal conductivity values of these felts at 30–1800 °C monotonically increased, with 0.29–3.02 W/m/K for PAN-based CF felt, 0.13–1.42 W/m/K for CF@CPBO-600 felt, 0.02–1.54 W/m/K for CF@CPBO-900 felt and 0.75–3.56 W/m/K for CF@CPBO-1400. Thermal conductivity values of these felts at a specific temperature followed the sequence CF@CPBO-1400 felt > PAN-based CF felt > CF@CPBO-900 felt ≥ CF@CPBO-600 felt. By taking the thermal conductivity values at 1200 °C as examples, the thermal conductivity of CF@CPBO-900 felt was 30.9% and 48.8% of those for CF@CPBO-1400 felt and PAN-based CF felt, respectively.

It is reported that the PBO-derived graphite layer on the outer layer of CF can significantly enhance the radial thermal conductivity of CF, thereby effectively improving the thickness-directional thermal conductivity of composite CF [25]. Due to the π-π interaction between PBO macromolecules and CF, the PBO layer can uniformly cover the surface of CF, inducing some PBO macromolecules to be oriented vertically [26]. During the high-temperature graphitization process, the large molecules of PBO can spontaneously form graphite crystals without any external force induction. The graphite crystals oriented perpendicularly to the surface of CF can provide a more effective heat conduction path between the CF, reducing the adverse effects of the interface thermal resistance [27]. Introducing PBO-derived carbon layers with a higher degree of graphitization on the fiber surface can increase the heat transfer path during the radial heat conduction of CF. The PBO-derived graphite layer has a greater thermal conductivity, thus having a higher phonon transmission efficiency. According to the theory of thermal elasticity of composite material heat conduction, the introduction of more high-thermal-conductivity components can weaken the adverse effect of the high-thermal-resistance resin matrix on the thermal conductivity of the composite material, thereby reducing the phonon scattering during heat conduction in the thickness direction of the composite material [28,29,30].

Therefore, from the perspective of heat insulation, it is necessary to avoid the formation of a PBO-derived graphite layer. Compared with PBO-derived graphite, PBO-derived amorphous carbon would be more appropriate to decrease the thermal conductivity of CF. In this study, CF@PBO was carbonized at 600 °C, 900 °C and 1400 °C, respectively. From both the XRD and Raman results, CF@CPBO-1400 presented a higher graphitization degree than those of CF@CPBO-600 and CF@CPBO-900. A low carbonization temperature (e.g., 600 °C and 900 °C) should be selected to avoid the high-temperature graphitization of PBO molecules. CF@CPBO-600 and CF@CPBO-900 would be promising candidates for encapsulating CF with reducing thermal conductivity. Moreover, considering the better mechanical properties of CF@CPBO-900 felt than CF@CPBO-600 felt, it is believed that CF@CPBO-900 felt presented the superior synergistic integration of mechanical and thermal conductivity properties. Compared with PAN-based CF, CF@CPBO-900 with decreased thermal conductivity and improved mechanical property has significant advantages in the aerospace field where both mechanical performance and thermal insulation are highly required.

## 4. Conclusions

In summary, PBO molecules were uniformly encapsulated onto the surface of air plasma-treated PAN-based CF. By increasing the carbonization temperature from 600 °C to 1400 °C, CF@CPBO presented a cleaner surface, more radial graphite layers within fibers, enhanced crystallinity of graphite layers (amorphous to 0.337 nm of interplanar spacing), reduced defective/graphitic content (0.959–0.909 of I_D_/I_G_), declined surface O content (20.1–9.6 at.%) and improved symmetry of the C-C peak. After weaving into a net and following compression molding, CF@CPBO-600 to CF@CPBO-1400 felts with random distributed structures (no voids and no fiber bundles) presented enhanced compression strength (145.3–127.9 MPa) at 60% of strain and excellent compression-recovery mechanical performance (130.9–110.8 MPa) through 10 cycles. Thermal conductivity values of these felts at 30–1800 °C monotonically increased, with 0.29–3.02 W/m/K for PAN-based CF felt, 0.13–1.42 W/m/K for CF@CPBO-600 felt, 0.02–1.54 W/m/K for CF@CPBO-900 felt and 0.75–3.56 W/m/K for CF@CPBO-1400. CF@CPBO carbonized at higher temperatures exhibited an increased mechanical property but a decreased thermal insulation property. Compared with a PBO-derived graphite layer, PBO-derived carbon produced at a lower carbonization temperature would be essential for achieving the optimal combination of mechanical and thermal-insulating properties. This work proposes an efficient strategy for regulating the high-performance organic fiber structure through heat treatment-induced processes.

## Figures and Tables

**Table 1:** Atomic ratios of C, O and N on the surface of samples.

	C (at.%)	O (at.%)	N (at.%)
PBO	73.7	23.9	2.4
PAN-based CF	81.0	16.6	2.4
CF@PBO	75.9	21.0	3.1
CF@CPBO-600	77.0	20.1	2.9
CF@CPBO-900	82.9	14.3	2.8
CF@CPBO-1400	85.7	9.6	4.7

## Data Availability

The original contributions presented in this study are included in the article. Further inquiries can be directed to the corresponding author.

## References

[1] Wu T., Lu C., Sun T., Li Y., Yuan S., Li D., Wang G., Ren X. (2022). New discovery on the relationship between microstructure and tensile strength of PAN-based carbon fibers. Microporous Mesoporous Mater..

[2] Montagna L.S., Ferreira de Melo Morgado G., Lemes A.P., Passador F.R., Rezende M.C. (2023). Recycling of carbon fiber-reinforced thermoplastic and thermoset composites: A review. J. Thermoplast. Compos. Mater..

[3] Yan C., Zhu Y., Liu D., Xu H., Chen G., Chen M., Cai G. (2023). Improving interfacial adhesion and mechanical properties of carbon fiber reinforced polyamide 6 composites with environment-friendly water-based sizing agent. Compos. Part B.

[4] Almushaikeh A.M., Alaswad S.O., Alsuhybani M.S., AlOtaibi B.M., Alarifi I.M., Alqahtani N.B., Aldosari S.M., Alsaleh S.S., Haidyrah A.S., Alolyan A.A. (2023). Manufacturing of carbon fiber reinforced thermoplastics and its recovery of carbon fiber: A review. Polym. Test..

[5] Pu Y., Ma Z., Liu L., Bai Y., Huang Y. (2022). Improvement on strength and toughness for CFRPs by construction of novel “soft-rigid” interface layer. Compos. Part B.

[6] Wang S., Haldane D., Gallagher P., Liu T., Liang R., Koo J.H. (2014). Heterogeneously structured conductive carbon fiber composites by using multi-scale silver particles. Compos. Part B.

[7] Sciti D., Zoli L., Vinci A., Silvestroni L., Mungiguerra S., Galizia P. (2021). Effect of PANbased and pitch-based carbon fibres on microstructure and properties of continuous Cf/ZrB2-SiC UHTCMCs. J. Eur. Ceram. Soc..

[8] Fang Z., Li M., Wang S., Gu Y., Li Y., Zhang Z. (2019). Through-thickness thermal conductivity enhancement of carbon fiber composite laminate by filler network. Int. J. Heat Mass Tran..

[9] Song G., Deng Q., Wang B., Liu Z., Ye C., Wei X., Dai D., Pan Z., Li W., Song S. (2020). Thermal and corrosion behavior of Ti_3_C_2_/Copper composites. Compos. Commun..

[10] Peng Y., Gong K., Liu A., Yan H., Guo H., Wang J., Guo X., Yang X., Qi S., Qiu H. (2025). Ultralight and rigid PBO nanofiber aerogel with superior electromagnetic wave absorption properties. J. Mater. Sci. Technol..

[11] Tang L., Jiang J., He M., Zhang Y., Hu Q., Liu X., Liu X., Qiu H. (2026). Lightweight PBO nanofiber@ZIF-67 derived carbon aerogel with superior electromagnetic wave absorption and thermal insulation. J. Mater. Sci. Technol..

[12] Brown K.R., Harrell T.M., Skrzypczak L., Scherschel A., Wu H.F., Li X. (2022). Carbon fibers derived from commodity polymers: A review. Carbon.

[13] Vázquez-Santos M.B., Geissler E., László K., Rouzaud J.N., Martinez-Alonso A., Tascon J.M.D. (2011). Comparative XRD, Raman, and TEM study on graphitization of PBO-derived carbon fibers. J. Phys. Chem. C.

[14] Wang C., Wang M., Chen Y., Liu L., Qin C., Huang Y. (2026). Poly(p-phenylene benzobisoxazole) fiber: Properties, applications, and advances in surface modification for improved interfacial and UV resistance. Adv. Compos. Hybrid Mater..

[15] Kaburagi Y., Yokoi K., Yoshida A., Hishiyama Y. (2010). Highly graphitized carbon fiber prepared from poly (p-phenylene-benzo-bis-oxazole) fiber. Tanso.

[16] Hao M., Hu Z., Huang Y., Qian X., Wen Z., Wang X., Liu L., Lu F., Zhang Y. (2022). Enhanced both in-plane and through-thickness thermal conductivity of carbon fiber/epoxy composites by fabricating high thermal conductive coaxial PAN/PBO carbon fibers. Compos. Part B.

[17] Pitto M., Fiedler H., Kim N.K., Verbeek C.J.R., Allen T.D., Bickerton S. (2024). Carbon fibre surface modification by plasma for enhanced polymeric composite performance: A review. Compos. Part A.

[18] Kilinc F.B., Bozaci E., Kilinc A.C., Turkoglu T. (2025). Effect of atmospheric plasma treatment on mechanical properties of 3D-printed continuous aramid fiber/PLA composites. Polymers.

[19] Wang S., Wang Y., Gao M., Huang Y. (2024). Aging effect of plasma-treated carbon fiber surface: From an engineering point. Coatings.

[20] Kosmachev P.V., Panin S.V., Panov I.L., Bochkareva S.A. (2022). Surface modification of carbon fibers by low-temperature plasma with runaway electrons for manufacturing PEEK-based laminates. Materials.

[21] Ayiania M., Smith M., Hensley A.J.R., Scudiero L., McEwen J.S., Garcia-Perez M. (2020). Deconvoluting the XPS spectra for nitrogen-doped chars: An analysis from first principles. Carbon.

[22] Zhang L., Kowalik M., Gao Z., Ashraf C.M., Rajabpour S., Bumgardner C., Schwab Y., Damirchi B., Zhu J., Akbarian D. (2020). Converting PBO fibers into carbon fibers by ultrafast carbonization. Carbon.

[23] Meng L., Sun G., Zhang T., Li H., Ye S. (2026). PBO-based carbon micro-nanofiber paper as catalyst carrier for fuel cell catalytic layer. Fuel.

[24] Liu Z., Song B., Wang T., Wang L. (2020). Significant improved interfacial properties of PBO fibers composites by in-situ constructing rigid dendritic polymers on fiber surface. Appl. Surf. Sci..

[25] Chen L., Du Y., Huang Y., Wu F., Cheng H.M., Fei B., Xin J.H. (2016). Hierarchical poly(*p*-phenylene benzobisoxazole)/graphene oxide reinforcement with multifunctional and biomimic middle layer. Compos. Part A.

[26] Zhao W., Kong J., Liu H., Zhuang Q., Gu J., Guo Z. (2016). Ultra-high thermally conductive and rapid heat responsive poly(benzobisoxazole) nanocomposites with self-aligned graphene. Nanoscale.

[27] Guo Y., Ruan K., Shi X., Yang X., Gu J. (2020). Factors affecting thermal conductivities of the polymers and polymer composites: A review. Compos. Sci. Technol..

[28] Yu H., Nonn A., Schneiders S., Heider D., Advani S.G. (2013). An approach to enhance through-thickness thermal conductivity of polymeric fiber composites. Int. J. Heat Mass Tran..

[29] Ruan K., Shi X., Guo Y., Gu J. (2020). Interfacial thermal resistance in thermally conductive polymer composites: A review. Compos. Commun..

[30] Li R., Yang X., Li J., Shen Y., Zhang L., Lu R., Wang C., Zheng X., Chen H., Zhang T. (2022). Review on polymer composites with high thermal conductivity and low dielectric properties for electronic packaging. Mater. Today Phys..

